# The Effects of Dietary Supplementation of *Lactococcus lactis* Strain Plasma on Skin Microbiome and Skin Conditions in Healthy Subjects—A Randomized, Double-Blind, Placebo-Controlled Trial

**DOI:** 10.3390/microorganisms9030563

**Published:** 2021-03-09

**Authors:** Ryohei Tsuji, Kamiyu Yazawa, Takeshi Kokubo, Yuumi Nakamura, Osamu Kanauchi

**Affiliations:** 1 Kirin Central Research Institute, Kirin Holdings Company, Ltd., Kanagawa 236-0004, Japan; Kamiyu_Yazawa@kirin.co.jp (K.Y.); Takeshi_Kokubo@kirin.co.jp (T.K.); 2 Department of Dermatology, Chiba University Graduate School of Medicine, Chiba 260-8670, Japan; yuminak312@gmail.com; 3 Cutaneous Immunology, Immunology Frontier Research Center, Osaka University, Osaka 565-0871, Japan; 4 Research and Development Strategy Department, Kirin Holdings Company, Ltd., Tokyo 164-0001, Japan; kanauchio@kirin.co.jp

**Keywords:** LC-Plasma, paraprobiotics, skin microbiome, *P. acnes*, *S. epidermidis*

## Abstract

(1) Background: *Lactococcus lactis* strain Plasma (LC-Plasma) is a unique strain which directly activates plasmacytoid dendritic cells, resulting in the prevention against broad spectrum of viral infection. Additionally, we found that LC-Plasma intake stimulated skin immunity and prevents *Staphylococcus aureus* epicutaneous infection. The aim of this study was to investigate the effect of LC-Plasma dietary supplementation on skin microbiome, gene expression in the skin, and skin conditions in healthy subjects. (2) Method: A randomized, double-blind, placebo-controlled, parallel-group trial was conducted. Seventy healthy volunteers were enrolled and assigned into two groups receiving either placebo or LC-Plasma capsules (approximately 1 × 10^11^ cells/day) for 8 weeks. The skin microbiome was analyzed by NGS and qPCR. Gene expression was analyzed by qPCR and skin conditions were diagnosed by dermatologists before and after intervention. (3) Result: LC-Plasma supplementation prevented the decrease of *Staphylococcus epidermidis* and *Staphylococcus pasteuri* and overgrowth of *Propionibacterium acnes*. In addition, LC-Plasma supplementation suggested to increase the expression of antimicrobial peptide genes but not tight junction genes. Furthermore, the clinical scores of skin conditions were ameliorated by LC-Plasma supplementation. (4) Conclusions: Our findings provided the insights that the dietary supplementation of LC-Plasma might have stabilizing effects on seasonal change of skin microbiome and skin conditions in healthy subjects.

## 1. Introduction

The skin is the largest epithelial layer and serves as a body shield by preventing evaporation of body fluids and protecting from the harmful environmental substances [1,2]. In addition to the skin’s own function as a first defense line against foreign substances, the skin is home to a diverse population of microbes. The majority of these skin microbes are nonpathogenic commensals or transients [3]. Epidermal commensal bacteria play an essential roles to maintain the healthy skin by preventing the colonization of pathogenic bacteria [4], regulating skin pH [5], and contributing to the immune response [6,7]. Since several skin disorders can be caused in case of the imbalance between commensal and pathogenic bacteria [8], the redundancy of skin microbiome is considered to be important to maintain the skin condition in healthy human.

*Propionibacterium acnes* (*P. acnes*) is one of the most dominant skin commensal bacteria. *P. acnes* has been thought to play a role in pathogenesis of acne vulgaris [9,10]. On the contrary, *P. acnes* has an ability to prevent the colonization of opportunistic pathogens by converting sebum to free fatty acid [11,12]. Since *P. acnes* has both beneficial and pathogenic roles to skin health, it is regarded as opportunistic bacteria. *Staphylococcus aureus* (*S. aureus*) is a ubiquitous bacterium on human skins and soft tissues. Persistent skin colonization is found in approximately 10–20% of healthy individuals and *S. aureus* infection often causes skin inflammations such as impetigo, folliculitis and cellulitis [13,14]. The traditional approach in most clinical laboratories for the differentiation between pathogenic *S. aureus* and other *Staphylococci* is the tube coagulase test [15]. In contrast to *S. aureus*, most species of *Staphylococcus* do not produce coagulase and thus are called as coagulase negative *Staphylococcus* (CoNS). The CoNS are not regarded as pathogens and their presence is suggested to influence *S. aureus* colonization [16,17,18,19]. In addition, *S. epidermidis* and other several strains of CoNS are reported to produce antimicrobial peptides (AMPs) and stimulate host skin immunity to induce AMPs [5,20]. Therefore, it is possible that *S. aureus* is regarded as pathogenic bacteria and CoNS, including *S. epidermidis*, is regarded as commensal bacteria on skin surface. Since genus *Propionibacterium* and genus *Staphylococcus* are known to be dominant and affect skin conditions among skin microbiome, it is important to assess the genus composition as well as skin condition.

*Lactococcus lactis* strain Plasma (hereinafter, LC-Plasma) is a synonym of *Lactococcus lactis* subsp. *lactis* JCM 5805. In the past reports, LC-Plasma was found as plasmacytoid dendritic cell (pDC) stimulative lactic acid bacteria [21]. LC-Plasma was reported to enhance antiviral immunity via pDC activation and prevent broad spectrum of viral infections, including respiratory, gastro-intestinal and vector-borne infections [22,23,24]. Recently, we reported that long-term administration of LC-Plasma prevented the skin thinning and the decrease of tight junction gene expressions in animal studies [25,26]. Furthermore, another animal study showed that LC-Plasma administration augmented skin immunity, resulting in the prevention of *S. aureus* epicutaneous infection and its related inflammation phenotypes [27]. Therefore, we hypothesized that LC-Plasma dietary supplementation may influence skin immunity and microbiome, then ameliorate skin phenotypes, which occurred by bacterial infection in human subjects as well.

In this study, we performed a double-blinded clinical trial using healthy subjects to focus on the effect of LC-Plasma dietary supplementation on skin microbiome, expression of skin condition-related genes, the skin conditions assessment and measurements of the trans-epidermal water loss (TEWL), skin moisture content and pigmentation values.

## 2. Materials and Methods

### 2.1. Ethical Standards

The clinical trial was conducted in accordance with the principles of the Declaration of Helsinki, and the Ethical Guidelines for Medical and Health Research Involving Human Subjects. The trial obtained approval from the Ethical Committee for the Oriental Ueno Detection Center, General Incorporated Association Oriental Occupational Health Association Tokyo Branch (no. HR-2018-KR06), then registered in the University Hospital Medical Information Network (UMIN) Clinical Trials Registry (no. UMIN000034061).

### 2.2. Subjects

The study enrolled healthy Japanese female participants (aged 20–<45 years). Participants received a sufficient explanation of this clinical trial and gave written informed consent before any inspections. Participants, who met inclusion criteria and did not meet exclusion criteria (Table 1), were selected as subjects of this trial through two selections. In the primary selection, 110 candidates were selected from 249 participants after screening on the basis of detection of *S. aureus* and/or high amount of *P. acnes* in skin microbiome. In the secondary selection, 70 subjects were selected from candidates after screening on the basis of high a-value, measured by colorimeter.

The minimum sample size was determined on the basis of CD86 expression, which is one of the activation markers of pDC, because pDC is suggested to be a direct target of LC-Plasma and immune-stimulatory effects locally, including skin, observed after pDC activation. Our past study reported that a standard deviation for CD86 was 20% and a difference in pre- and postadministration mean values was 15% [28]. According to these parameters, we calculated that a sample size of 29 subjects in each arm would be required to achieve at least 80% power (β ≥ 0.8) with statistical significance (α ≤ 0.05) in a paired *t*-test.

### 2.3. Study Design, Outcomes, Randomization and Allocation

The present study was designed as a randomized, double-blind, placebo-controlled, parallel-group trial to evaluate the effects of LC-Plasma supplementation on skin microbiome and skin condition in healthy Japanese females. The primary outcomes of this study were the change of skin microbiome composition on forehead and the expression level of skin condition-related genes in hair follicles. The secondary outcomes were diagnosis of skin condition by dermatologists, pigmentation scores (including L*a*b values), skin moisture and trans-epidermal water loss (TEWL). Eligible subjects were randomly allocated to either the placebo group (*n* = 35) or the LC-Plasma group (*n* = 35) using a stratified block randomization considering age, BMI, a-values which were measured in secondary screening, and number of *S. aureus* positive subjects and *P. acnes* counts which were measured in primary screening. Subjects in the LC-Plasma group consumed one hard capsule, containing 50 mg (approximately 1 × 10^11^ cells) of heat-killed and spray-dried LC-Plasma (Kyowa Hakko Bio Co. Ltd., Tokyo, Japan) per day and subjects in the placebo group consumed the same capsule, not containing genetically modified cornstarch instead of LC-Plasma, per day for 8 weeks from October to December 2018. Neither the subjects nor the investigators could distinguish visually between the capsules. The allocation table was sealed and stored securely by the controller. The capsule codes were blinded, and the data set was locked until all analyses were completed. Subjects were instructed to visit hospitals on week 0 (baseline) and week 8 (after ingestion).

### 2.4. Collection of Skin Microbiome

Either showering or bathing were not permitted for 12 h before sample collection. Skin swab samples were obtained from above eyebrow by swabbing the skin for 30 s in a 2 × 4 cm area. DNA was extracted from swab samples with the QIAamp UCP Pathogen Mini Kit (Qiagen, Hilden, Germany) according to the manufacturer’s instruction. Elution volume was fixed for 100 μL, then DNA solutions were used for next-generation sequencer analysis and quantitation for *P. acnes*, *S. epidermidis* and *S. aureus*, described below.

### 2.5. Next-Generation Sequencer Analysis

After DNA extraction, seven bacterial 16S hypervariable regions were amplified by polymerase chain reaction with following two primer sets: one for the V2-4-8 hypervariable regions and the other for the V3-6, 7-9 hypervariable regions. The primers were obtained from the Ion 16S^TM^ Metagenomics Kit (Thermo Fisher Scientific, Grand Island, NY, USA). The amplified fragments were barcoded and sequenced on the Ion PGM sequencer system (Thermo Fisher Scientific) using 318 chips with the Ion PGM Hi-Q View Sequencing Kit (Thermo Fisher Scientific) in accordance with the manufacturer’s instruction. Low-quality reads and short sequence reads (less than 120 bp) were excluded. The 16S rRNA sequences were subjected to homology searches for a BLAST analysis against the NCBI 16S Microbial database (National Center for Biotechnology Information, Bethesda, Rockville, MD, USA), and classified using Metagenome@KIN (World Fusion, Tokyo, Japan). The sequences with an E-value of >0.001 were clustered as “No BLAST hit”, and those with an E-value of >10^−30^ and OTU of <95% identity were clustered as “Unclassified hit”. All the processes of DNA extraction, PCR amplification, next-generation sequencing, and bioinformatics analysis were performed by World Fusion, Co. Ltd. (Tokyo, Japan).

### 2.6. Quantitation of Representative Bacteria and Calculation of Total Bacteria

The amounts of *P. acnes*, *S. epidermidis* and *S. aureus* were measured by performing qRT-PCR using the genesig standard kit of each bacteria (Primerdesign, Camberley, UK). *P. acnes* JCM 6425, *S. epidermidis* NBRC 12993, and *S. aureus* MW2 were used as positive controls of each bacteria and calculated as CFU equivalent. Total bacterial counts were calculated by dividing the counts by the composition ratio of *P. acnes*.

### 2.7. Gene Expression in Hair Follicle

Total RNA samples from scalp hair follicles were isolated as previously described with some modification [29]. Briefly, 5–10 hairs were plucked from the scalp of each subject. After confirming the presence of the scalp, hairs were trimmed to approximately 1.0 cm to collect the bulb region, and dipped into RNAlater solution (Ambion, Grand Island, NY, USA), then stored at 4 °C until RNA extraction. Total RNA was extracted using the RNAqueous-4PCR Kit (Invitrogen, Waltham, MA, USA) in accordance with manufacturer’s instruction after removing RNAlater solution.

cDNA was synthesized by using an iScript cDNA synthesis kit (BioRad, Hercules, CA, USA), by following the manufacturer’s protocol. qRT-PCR was performed using SYBR Premix EX Taq (TaKaRa, Tokyo, Japan) using a LightCycler 480 (Roche, Mannheim, Germany). *Gapdh* was used as the reference gene. The amount of expression of each gene was calculated with using relative standard curve method [30]. The primer sequences used in this study are listed in Table 2.

### 2.8. Diagnosis of Skin Condition by Dermatologists

Skin conditions of subjects were diagnosed by dermatologists before and after administration of study products. The severity of dryness, erythema, scale, irritation, and itching were assessed from five grades: (0) normal, (1) slight, (2) mild, (3) moderate, and (4) severe.

### 2.9. Skin Moisture and Trans-Epidermal Water Loss (TEWL), Pigmentation Assessment

Before measurement of skin condition, the subjects washed their faces using a prescribed cleansing foam and then spent 20 min becoming accustomed to the environment of the measurement room at a constant temperature and humidity (room temperature, 21 ± 1 °C; relative humidity, 50 ± 10%). Skin moisture in the stratum corneum was measured using a Corneometer^®^ CM825 instrument (Courage + Khazaka Electronic GmbH, Cologne, Germany) and trans-epidermal water loss (TEWL) scores (g/h/m^2^) were obtained by using a Tewameter^®^ TM300 instrument (Courage + Khazaka Electric GmbH). Pigmentation of the skin equivalents was assessed by comparing the change in L*, a*, and b* value, values of CIE 1976 color space. The L* value represents the brightness, the a* value represents the green–red component and the b* value represents the blue–yellow component. All equivalents were measured using CM-2600d (Konika minorta, Tokyo, Japan). The measurement sites were set at the bone area in left cheek and each value was determined as the average of three measurements.

### 2.10. Blood Collection and Safety Evaluation

Serum was obtained from all subjects before and after administration of study products to perform general biochemical examination of blood, including total protein, albumin, urea nitrogen, creatinine, creatine kinase, uric acid, aspartate aminotransferase, alanine transaminase, γ-glutamyl transpeptidase, alkaline phosphatase, lactate dehydrogenase, total bilirubin, C-reactive protein, total cholesterol, low-density lipoprotein cholesterol, high-density lipoprotein cholesterol, triglyceride, sodium, potassium, chloride, glucose, and HbA1c. Blood was obtained to perform hematologic tests, including white blood cell, red blood cell, hemoglobin, hematocrit, and platelet. These analyses were performed for safety evaluation.

### 2.11. Statistical Analyses

Nonpaired *t*-tests were used for the allocation of subjects into the placebo group and the LC-Plasma group. Comparisons of continuous variables of all parameters on week 0 and week 8 in each group were performed using the paired *t*-test (parametric) or Wilcoxon Signed-Rank test (nonparametric). A significant difference was defined as *p* < 0.05 and moderate difference was defined as *p* < 0.1. For analysis of skin microbiome, the data of “Unclassified hit”, “No BLAST hit” and those whose average compositions of either group at week 0 are less than 0.1% were excluded from statistical analysis to remove the noises. Comparisons of continuous variables on week 0 and week 8 were performed using the Wilcoxon Signed-Rank test and to correct for multiple comparisons, *q* values were calculated with the Benjamini–Hochberg false discovery rate (FDR) correction as a post-hoc test. A significance difference was defined as *q* < 0.05 and moderate difference was defined as *q* < 0.1. All statistical analyses were performed with R software (ver. 3.6.2). Nonpaired *t*-tests were not adopted in this study due to the large variations in skin microbiome composition and gene expression levels among participated subjects.

## 3. Results

### 3.1. Subject Characteristics

**Figure 1:**
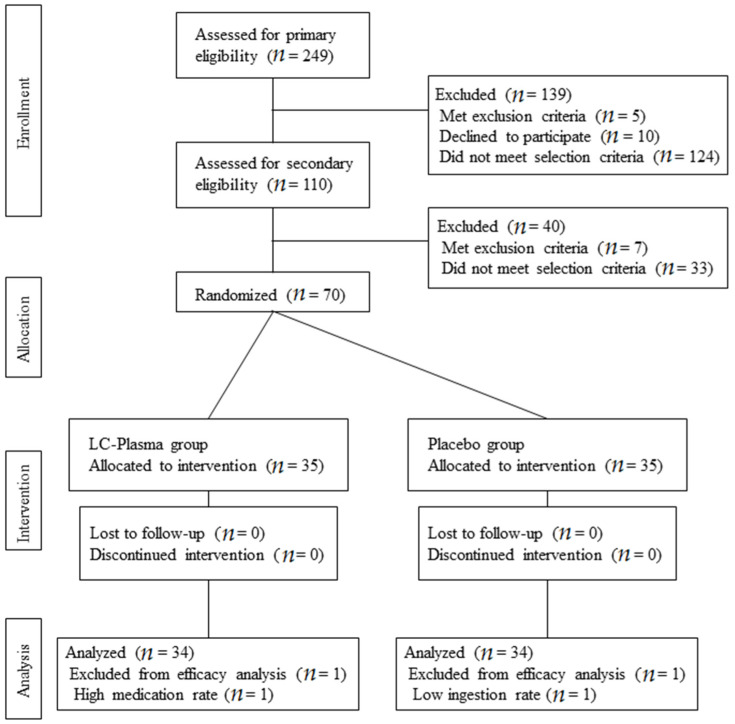
Flow diagrams of subjects.

The consolidated standards of flow diagram for this study are shown in Figure 1. In total, 249 candidates were recruited and 70 subjects were enrolled through primary and secondary selection as described in Section 2. Eligible subjects were randomly allocated into two groups: placebo group (*n* = 35) and LC-Plasma group (*n* = 35). No subjects were lost to follow-up during the intervention period. One subject in the placebo group and one subject in the LC-Plasma were excluded from analysis due to the low ingestion ratio (<85%) and high medication rate (>35%). Finally, 34 subjects in the placebo group and 34 subjects in the LC-Plasma group were analyzed to evaluate the efficacy of LC-Plasma supplementation. The baseline conditions of analyzed subjects are shown in Table 3.

### 3.2. The Change of Composition of Skin Microbiome

**Figure 2:**
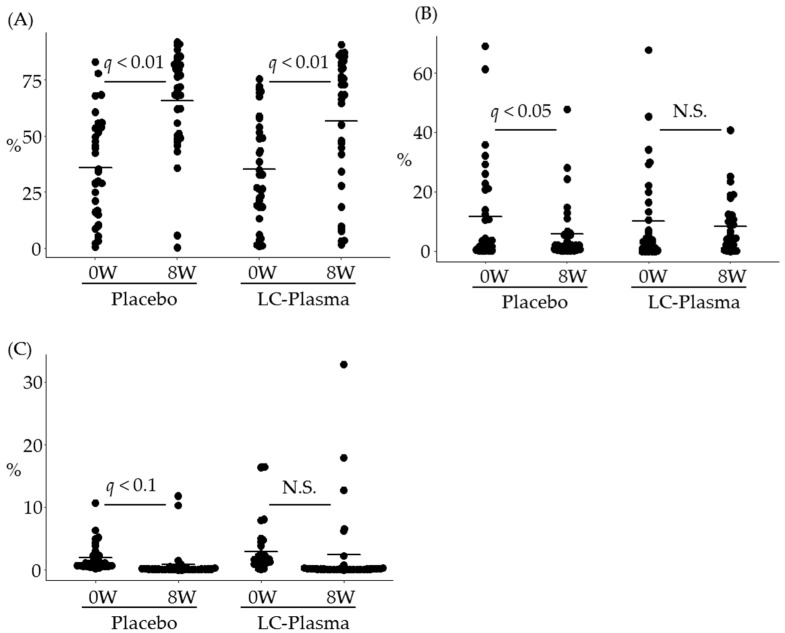
Prevention of the decrease of genera *Staphylococcus* and *Sphingomonus* composition ratio by (*Lactococcus lactis* strain Plasma) LC-Plasma supplementation. The composition ratio of representative genus was calculated from NGS analysis, then compared with continuous variables between weeks 0 and 8. (**A**) The composition ratio of genus *Propionibacterium*. (**B**) The composition ratio of genus *Staphylococcus*. (**C**) The composition ratio of genus *Sphingomonus*. The short line in all figures represents the mean value. Paired Student’s *t*-test between weeks 0 and 8 was performed both in the placebo group and the LC-Plasma group, then corrected to control FDR and calculated as *q* values. *q* values with less than 0.05 were defined as significantly different and *q* values with less than 0.1 were defined as moderately different. N.S. means not significant in this figure.

**Figure 3:**
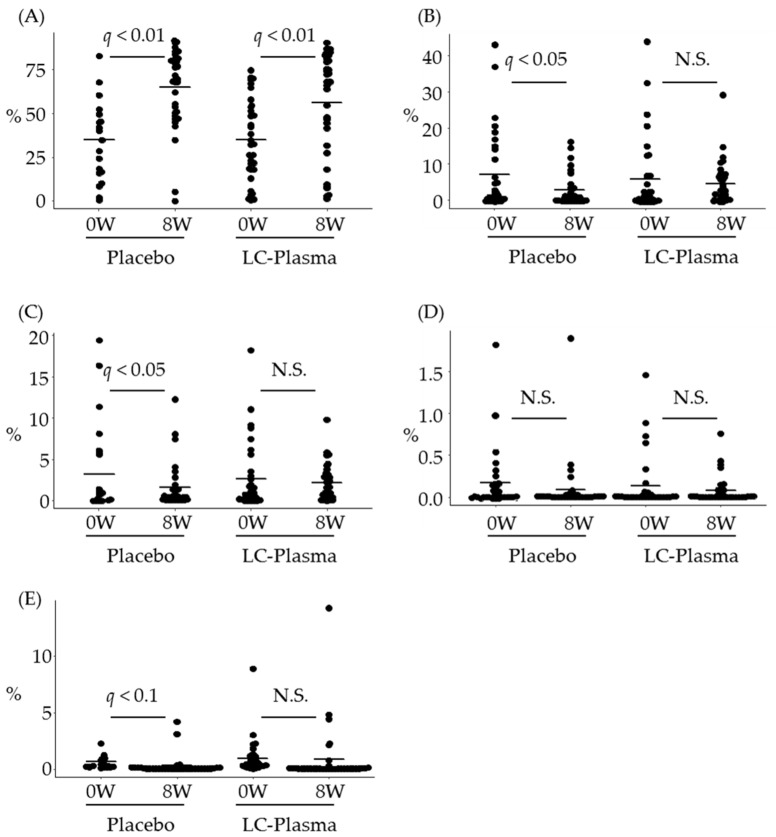
Prevention of the decrease of *Staphylococcus epidermidis*, *Staphylococcus pasteuri* and *Sphingomonus roseiflava* composition ratio by LC-Plasma supplementation. The composition ratio of representative species was calculated from NGS analysis, then compared with continuous variables between weeks 0 and 8. (**A**) The composition ratio of *Propionibacterium acnes*. (**B**) The composition ratio of *S. epidermidis*. (**C**) The composition ratio of *S. pasteuri*. (**D**) The composition ratio of *Staphylococcus aureus*. (**E**) The composition ratio of *S. roseiflava*. The short line in all figures represents the mean value. Paired Student’s *t*-test between weeks 0 and 8 was performed both in the placebo group and the LC-Plasma group, then corrected to control FDR and calculated as *q* values. *q* values with less than 0.05 or 0.01 were defined as significantly or highly significantly different and *q* values with less than 0.1 were defined as moderately different. N.S. means not significant in this figure.

To evaluate the effect of LC-Plasma on skin microbiome, bacterial samples were collected from above the eyebrow area. Bioinformatics analysis was performed, and the microbiome data were classified into bacterial taxa, from phyla to species. The data of “Unclassified hit”, “No BLAST hit” and the low composition ratio (less than 0.1%) in either group at week 0 were excluded for further statistical analysis. As a result, 77 genera and 90 species were analyzed to evaluate the effect of LC-Plasma supplementation. The change of microbiome composition ratio was compared between week 0 (baseline) and week 8 (after ingestion) in each group by performing paired *t* tests and FDR correction as a post-hoc test. In genus level, as shown in Figure 2A, surprisingly, the composition ratio of genus *Propionibacterium*, which is the most abundant genus, was extremely increased in both groups (*q* < 0.01, in both groups). On the other hand, that of genus *Staphylococcus*, which is the second most abundant genus, was significantly decreased only in the placebo group (*q* = 0.04) and not changed in the LC-Plasma group (*q* = 0.61) (Figure 2B). The composition ratio of genus *Sphingomonus*, the third most abundant genus, was moderately decreased in the placebo group (*q* = 0.08), while that in the LC-Plasma group was not (*q* = 0.73) (Figure 2C). In species level, the composition ratio of *Propionibacterim acnes* was extremely increased in both groups as well as genus level (*q <* 0.01, in both groups) (Figure 3A). The ratios of *Staphylococcus epidermidis* and *S. pasteuri* were significantly decreased only in the placebo group (*q* = 0.02 for *S. epidermidis* and *q* = 0.03 for *S. pasteuri*, respectively) (Figure 3B,C), while those in the LC-Plasma group were not changed (*q* = 0.51 for *S. epidermidis* and *q* = 0.55 for *S. pasteuri*, respectively) (Figure 3B,C). On the other hand, the composition ratios of *S. aureus*, *S. capitis*, and *S. hominis* were not changed in both groups (Figure 3D and data not shown). Since the mean values of ratios of these species were very low (less than 0.2%), it seems to be difficult to observe the obvious changes of these species. Additionally, the composition ratio of *Sphingomonus roseiflava*, the most abundant species in genus *Sphingomonus*, was moderately decreased in the placebo group (*q* = 0.06), while that in the LC-Plasma group was not changed (*q* = 0.93). These data suggested that, interestingly, dietary supplementation of LC-Plasma led the prevention of the decrease of nonpathogenic species of *Staphylococcus* and *Sphingomonus* in the cutaneous layer.

### 3.3. The Change of Counts of Total Bacteria and Representative Species

**Figure 4:**
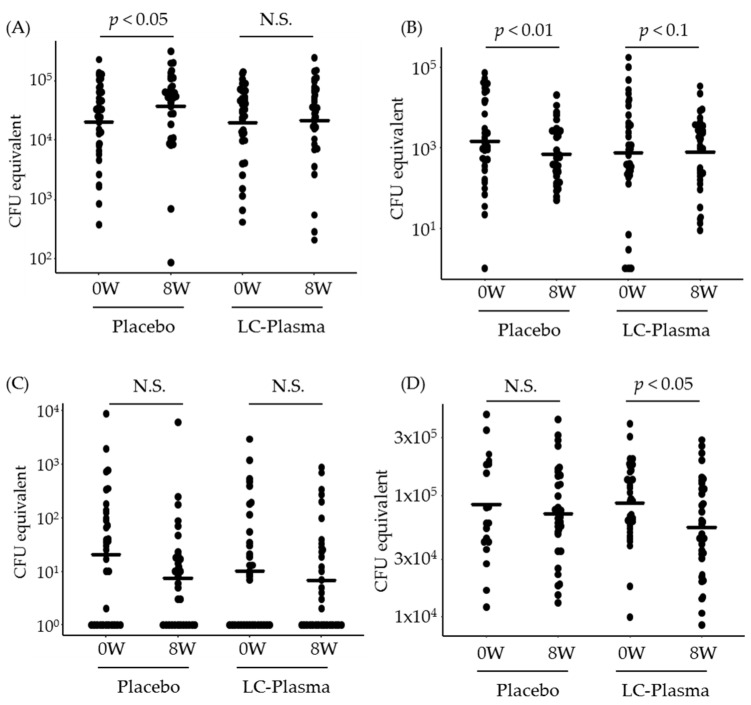
Prevention of the increase of *P. acnes* bacterial counts by LC-Plasma supplementation. Bacterial counts were quantified by qPCR, then calculated as CFU equivalent. The bacterial counts were compared with continuous variables between week 0 and week 8. (**A**) The counts of *P. acnes*. (**B**) The counts of *S. epidermidis*. (**C**) The counts of *S. aureus*. (**D**) The counts of total bacteria. The total bacterial counts were calculated by dividing the counts by the composition ratio of *P. acnes*. Paired Student’s *t*-test between weeks 0 and 8 was performed both in the placebo group and the LC-Plasma group. *p* values with less than 0.05 or 0.01 were defined as significantly or highly significantly different and *p* values with less than 0.1 were defined as moderately different. N.S. means not significant in this figure.

Next, we measured the bacterial counts of *P. acnes, S. aureus* and *S. epidermidis* by quantitative PCR, then calculated CFU equivalent with using the positive control of each species. Additionally, the total bacterial counts were also calculated from the data of *P. acnes* CFU equivalent and *P. acnes* composition ratio. Surprisingly, the counts of *P. acnes* were significantly increased in only the placebo group (*p* = 0.03 in the placebo and *p* = 0.29 in the LC-Plasma group) (Figure 4A). On the contrary, those of *S. epidermidis* were significantly decreased in the placebo group (*p* < 0.01), while those in the LC-Plasma group were moderately decreased (*p* = 0.07) (Figure 4B). The counts of *S. aureus* were not significantly changed in both two groups (Figure 4C). As shown in Figure 4D, total bacterial counts in the LC-Plasma group were significantly decreased after intervention period (*p* = 0.02), while those in the placebo group were not changed (*p* = 0.31). These data suggested that LC-Plasma dietary supplementation might mildly suppress the overgrowth of *P. acnes* and the decrease of *S. epidermidis*.

### 3.4. Alpha Diversity of Skin Microbiome

Additionally, the indexes of alpha diversity (Simpson and Shannon indexes) were calculated at genus and species levels. As shown in Table 4, the mean values of Simpson indexes of genus and of species were significantly decreased both in the placebo group and the LC-Plasma group at week 8 compared with those at week 0. The mean values of Shannon indexes were also significantly decreased in both groups. The significant decrease of alpha-diversity was due to the extreme increase of the composition ratio of *P. acnes*, which is the most abundant bacteria in skin microbiome, as described above. These data suggested that LC-Plasma administration cannot change the diversities of skin microbiome, but may prevent the overgrowth of specific bacterial species.

### 3.5. The Change of Gene Expressions in Hair Follicles

Since oral administration of LC-Plasma has been confirmed to stimulate skin immunity and induce the expressions of cytokines, antimicrobial peptide (AMP) and tight junction (Tj) genes in preclinical study [27], we investigated the effects of LC-Plasma dietary supplementation on gene expression levels in hair follicles as a representative skin tissue. As shown in Table 5, *TGFB1* gene was significantly upregulated in the LC-Plasma group (*p* = 0.02) but not changed in the placebo group (*p* = 0.20). Among the representative AMP genes, *BD3* expression was significantly increased in the LC-Plasma group (*p* < 0.01) but not in the placebo group (*p* = 0.63), and *S100A8* expression was moderately increased in the LC-Plasma group as well (*p* = 0.06 in the LC-Plasma group and *p* = 0.26 in the placebo group). The expressions of *BD1* and *S100A9* were significantly increased in both groups during the intervention period. *BD2* expression was not statistically changed in both groups. Among Tj genes, the expressions of *Cldn1* and *OCLN* were significantly upregulated in both groups, and *ZO1* expression was not changed in both groups. Interestingly, the expression of *OVOL-1* was significantly downregulated in the placebo group (*p =* 0.03), but not in the LC-Plasma group (*p* = 0.54).

### 3.6. Diagnosis of Skin Conditions

The skin conditions of representative five symptoms were diagnosed and scored by dermatologists. As shown in Table 6, the scores of dryness were significantly increased in the placebo group (*p* < 0.01) but not in the LC-Plasma group (*p* = 0.50). On the contrary, the scores of erythema were significantly decreased in the LC-Plasma group (*p* = 0.04) but not in the placebo group (*p* = 0.11). The scores of scale were not significantly changed in both groups. The symptoms of irritation and itching were rarely observed from subjects in this clinical trial, and the scores were not significantly changed in both groups.

### 3.7. Measurement of Skin Conditions

For the assessment of skin conditions, TEWL, skin moisture contents and pigmentation were measured, and results are shown in Table 7. TEWL was significantly increased in both groups. Skin moisture contents was not statistically changed but the mean values were decreased in both groups. The values of L* and HbSO_2_ were significantly increased in both groups, while the values of b* and Melanin indices were significantly decreased in both groups. Value of a*, and Hb were not changed in both groups. These data suggested that the seasonal changes of skin conditions could be detected in this clinical trial, but the difference between two groups could not be found.

### 3.8. Safety Evaluation

We observed no severe adverse events or changes in the scores of biochemical analysis in serum and hematological examination in blood (data not shown). Additionally, the adverse effects related to the administration of LC-Plasma were not observed. Therefore, clinical physicians concluded that there were no significant adverse effects related to LC-Plasma supplementation in this study.

## 4. Discussion

Even though the classical probiotics, defined as alive microorganisms, proved to provide health benefits to the hosts, some studies have proved that inactivated probiotic microorganisms can also provide such benefits. Then, these inactivated probiotic microorganisms were called as “paraprobiotics” [31,32]. In our previous studies, oral administration of heat-killed LC-Plasma activated pDCs and prevented viral infections in both animal and clinical studies [21,33]. Furthermore, LC-Plasma was proved to activate pDCs in skin-draining lymph nodes, resulting in protection from *S. aureus* epicutaneous infection [27]. In addition, long-term administration of LC-Plasma was reported to affect skin conditions in senescence-accelerated mice [25,26]. However, the effects on skin condition to human subjects is still unknown. Thus, we aimed to confirm the effects of LC-Plasma dietary supplementation on skin conditions in healthy human subjects.

Since *S. aureus* skin infection often causes skin inflammations and overgrowth of *P. acnes* in skin also causes skin inflammations such as acne vulgaris [13,34], among the healthy volunteers, we selected subjects of *S. aureus* detectable or high counts of *P. acnes* in skin and high a* values, which is one of the indications of erythema [35]. According to the NGS analysis, the tendency of skin microbiome diversity change was almost same between the placebo group and the LC-Plasma group (Table 4). The significant decrease of alpha-diversities in both groups was caused by the significant increase of *P. acnes* composition rate (Figure 3A). On the other hand, from the results of quantitation of representative skin bacteria, interestingly, the counts of *P. acnes* were not significantly increased and those of *S. epidermidis* were not significantly decreased only in the LC-Plasma group compared with the start date of intervention (Figure 4A,B). In previous study, *S. epidermidis* functioned as inhibitors of *P. acnes* overgrowth [36]. Therefore, the maintenance of *S. epidermidis* composition by LC-Plasma supplementation suggested to prevent the overgrowth of *P. acnes*. In addition to *S. epidermidis*, the ratio of *S. pasteuri* was also not significantly decreased in the LC-Plasma group (Figure 3C). *S. pasteuri* was classified as CoNS [37] and frequently isolated from clinical specimens, including skin and food [38]. The physiological functions of skin donated *S. pasteuri* was not well investigated, but it is reported that *S. pasteuri* can produce antimicrobial substances against *S. aureus* [39]. In this study, we could not observe statistical change of *S. aureus* in both composition ratio and CFU equivalent (Figure 3D and Figure 4C). According to the previous report of LC-Plasma, we expected the preventive effects on *S. aureus* overgrowth or its related symptoms in human subjects, but the baseline dose of *S. aureus* was lower than we expected to evaluate the effects in this trial. Therefore, we should perform further trials using *S. aureus* suffered patients to clarify the effects on *S. aureus* skin infection.

From the data of AMP genes expression, three genes out of five were significantly or moderately increased only in the LC-Plasma group (Table 5). Therefore, totally speaking, LC-Plasma dietary supplementation might lead to AMP induction in skin, resulting in the significant decrease of total bacteria in skin and significant decrease of “erythema” scores (Table 6). On the other hand, the a* values of skin were not different between two groups (Table 7). The a* values were measured in limited area, left cheek bone area, while dermatologists provided scores after diagnosing whole faces. Therefore, we suggested that this was the reason why the tendency of erythema scores and a* values were different.

Interestingly, OVOL1 expression was significantly decreased only in the placebo group (Table 5). OVOL1 was reported to express in the nuclei of germ cells in hair bulbs and related with hair differentiation [40]. In addition, OVOL1 is known to express in keratinocytes and its expression is strongly suggested to have an important role in the formation of skin barrier functions [41,42]. These studies suggested the reason why the scores of “dryness” were significantly increased only in the placebo group (Table 6). However, since TEWL and water contents were not different between the two groups and these two indices were deteriorated due to the seasonal changes that are usually observed in autumn in Japan (Table 7). In addition, the tendency of change of Tj genes was not different between the two groups. Therefore, we should perform further investigations to clarify the potency to affect the skin barrier function by LC-Plasma supplementation in future.

Our study has several limitations. In this study, we had to enroll *S. aureus* negative subjects due to the low ratios of *S. aureus* positive (45 out of 251 candidates) in healthy people as previously reported [43]. In addition, we enrolled the healthy subjects without any specific symptoms in skin and randomly allocated with mechanical values. However, unfortunately, the scores of skin conditions, diagnosed by dermatologists, were highly different at the start date of intervention. Therefore, it was suggested to be difficult to detect the obvious effects of LC-Plasma on skin conditions. We performed the NGS analysis for skin microbiome analysis from phylum to species level. However, the pathogenesis of acne vulgaris were suggested to be related with the ribotypes of *P. acnes* [44]. Thus, we should further investigate the effects of LC-Plasma supplementation of the change of *P. acnes* compositions in ribotypes level. We measured the a* value which can be a useful indicator of skin inflammation [35], but we could not distinguish the causes of inflammation among infection of pathogenic microorganisms, allergy responses, UVB exposure, and so on. According to these limitations, we need to perform further clinical trials to confirm the effects of LC-Plasma on subjects with erythema skin, including UV-B exposed healthy subjects, patients who have obvious clinical symptoms, caused by skin colonization of pathogenic bacteria, in order to minimize the seasonal factors.

## 5. Conclusions

In summary, we can provide the original effects of heat-killed LC-Plasma dietary supplementation on seasonal change of skin microbiome and deterioration of skin conditions in healthy human subjects. In addition to the recent report that probiotics can modulate skin microbiome composition [45], our findings provided new interesting insights into the regulation of skin microbiome by paraprobiotics. According to the latest publication, the skin microbiome was linked to the health-related skin phenotypes, including skin hydration [46], thus the regulation of skin microbiome by paraprobiotics affects host skin health via host–microbiome interaction.

## Figures and Tables

**Table 1:** Inclusion and exclusion criteria in this trial.

**Inclusion Criteria**	
(1)	Healthy Japanese females aged from 20 to less than 45 years old, when giving the informed consent.
(2)	Healthy individuals not having any chronic disease.
(3)	Individuals whose skin bacteria can be detected from forehead.
(4)	Individuals who generally have skin problems.
(5)	Individuals who are working more than 3 days per week.
(6)	Individuals who give the informed consents in writing, after receiving enough explanation of the purpose and details of the study, understanding the study well, and deciding to attend the study with their own will.
(7)	Individuals who can accomplish their tasks in the study at the appointed date.
(8)	Individuals who are judged suitable for this study by the investigators.
**Exclusion Criteria**	
(1)	Individuals who have diseases with medications.
(2)	Individuals who receive medications within 1 month before examination.
(3)	Individuals who have a medical history of serious disease of liver, kidney, heart, lung, blood and digestive tract.
(4)	Individuals who have severe skin disorder, such as skin burn.
(5)	Individuals who are difficult to take samples for gene expression analysis.
(6)	Individuals who refuse to disclose their biological sexes.
(7)	Individuals who may have an allergic symptom to test foods, or individuals who may have a serious allergic symptom to other foods, or medicaments.
(8)	Individuals who are alcoholic or have mental disorders.
(9)	Individuals who have a smoking habitat.
(10)	Individuals who will change their life style during test period, such as traveling for a long period.
(11)	Individuals who cannot keep from direct sunlight exposure, such as tanning activities, during test period.
(12)	Individuals who may occur seasonal allergic symptoms, such as hay fever, and receive medications during test period.
(13)	Individuals who have severe menopausal symptoms.
(14)	Individuals who are taking or took foods or medications, specified for skin conditions or are planning to take these foods during test period.
(15)	Individuals who cannot stop eating probiotics or lactic acid bacteria containing foods during test period.
(16)	Individuals who have severe anemia.
(17)	Individuals who donate more than 200 mL of blood within 1 month or more than 400 mL of blood within 3 months.
(18)	Individuals who have a surgical or treatment history on the regions of measurement within 6 months.
(19)	Individuals who are pregnant, breastfeeding, or planning to be pregnant in the near future.
(20)	Individuals who are participating or participated in another clinical trial within the last 3 months.
(21)	Individuals who and whose family living with them work for a company manufacturing or selling healthy foods or cosmetics.
(22)	Individuals who are judged as unsuitable for participating this study by the investigator.

**Table 2:** Primer sequences for qRT-PCR.

Gene		Primer Sequence
*TGFB1*	F′	5′-CCCAGCATCTGCAAAGCTC-3′
	R′	5′-GTCAATGTACAGCTGCCGCA-3′
*BD1*	F′	5′-ACCTTCTGCTGTTTACTCTCTGCTTAC-3′
	R′	5′-TCCACTGCTGACGCAATTGTA-3′
*BD2*	F′	5′-TCCTCTTCTCGTTCCTCTTCATATTC-3′
	R′	5′-GACTGGATGACATATGGCTCCAC-3′
*BD3*	F′	5′-CCATTATCTTCTGTTTGCTTTGCTC-3′
	R′	5′-CCGCCTCTGACTCTGCAATAATA-3′
*S100A8*	F′	5′-ATGCCGTCTACAGGGATGAC-3′
	R′	5′-ACGCCCATCTTTATCACCAG-3′
*S100A9*	F′	5′-TCATCAACACCTTCCACCAA-3′
	R′	5′-GTGTCCAGGTCCTCCATGAT-3′
*CLDN1*	F′	5′-CTGCCCCAGTGGAGGATTTA-3′
	R′	5′-CATGGCCTGGGCGGT-3′
*ZO1*	F′	5′-CAGCCGGTCACGATCTCCT-3′
	R′	5′-TCCGGAGACTGCCATTGC-3′
*OCLN*	F′	5′-AACCCAACTGCTCAGTCTTC-3′
	R′	5′-TGATCCACGTAGAGTCCAGTAG-3′
*OVOL1*	F′	5′-CCGTGCGTCTCCACGTGCAA-3′
	R′	5′-GGCTGTGGTGGGCAGAAGCC-3′
*GAPDH*	F′	5′-GCACCGTCAAGGCTGAGAAC-3′
	R′	5′-TGGTGAAGACGCCAGTGGA-3′

**Table 3:** Background values of the analyzed subjects.

Item	Placebo	LC-Plasma	*p*-Value
Number of subjects	34	34	
Age	34.1 ± 7.2	34.0 ± 6.5	0.94
BMI	21.1 ± 2.9	20.6 ± 2.5	0.508
*P. acnes* counts (CFU equivalent)	39172 ± 37324	45784 ± 51079	0.55
Number of *S. aureus* positive subjects	21/34	24/34	
a-value	7.72 ± 1.41	7.90 ± 1.62	0.51

**Table 4:** The change of skin microbiome diversity.

		Simpson Index			Shannon Index		
		0W	8W	*p* Value	0W	8W	*p* Value
Genus	Placebo	0.72 ± 0.19	0.47 ± 0.22	0.000 **	3.61 ± 1.36	2.03 ± 1.14	0.000 **
	LC-Plasma	0.71 ± 0.19	0.52 ± 0.26	0.000 **	3.47 ± 1.22	2.26 ± 1.31	0.000 **
Species	Placebo	0.76 ± 0.19	0.49 ± 0.23	0.000 **	4.45 ± 1.64	2.51 ± 1.49	0.000 **
	LC-Plasma	0.74 ± 0.19	0.55 ± 0.28	0.000 **	4.22 ± 1.44	2.91 ± 1.75	0.000 **

Mean ± S.D. **: *p* < 0.01.

**Table 5:** Relative expression levels of skin homeostasis related genes.

Indexes	Group	0W	8W	*p* Value
Cytokine genes				
*TGFB1*	Placebo	0.86 ± 0.19	0.92 ± 0.28	0.192
	LC-Plasma	0.93 ± 0.24	1.05 ± 0.33	0.019 *
AMP genes				
*BD1*	Placebo	0.86 ± 0.59	1.13 ± 0.79	0.042 *
	LC-Plasma	1.12 ± 1.02	1.35 ± 1.02	0.02 *
*BD2*	Placebo	0.77 ± 1.24	1.32 ± 2.48	0.134
	LC-Plasma	0.61 ± 1.42	0.91 ± 1.96	0.225
*BD3*	Placebo	0.79 ± 0.70	0.83 ± 0.59	0.628
	LC-Plasma	0.76 ± 0.55	0.97 ± 0.59	0.008 **
*S100A8*	Placebo	0.74 ± 0.49	0.86 ± 0.66	0.257
	LC-Plasma	0.86 ± 0.68	1.14 ± 0.97	0.063 ^†^
*S100A9*	Placebo	0.87 ± 0.44	1.05 ± 0.40	0.023 *
	LC-Plasma	1.08 ± 0.58	1.33 ± 0.66	0.007 **
TJ genes				
*Cldn1*	Placebo	0.86 ± 0.30	1.05 ± 0.40	0.000 **
	LC-Plasma	1.06 ± 0.41	1.47 ± 0.53	0.000 **
*OCLN*	Placebo	0.85 ± 0.31	1.08 ± 0.38	0.003 **
	LC-Plasma	0.98 ± 0.36	1.18 ± 0.43	0.026 *
*ZO1*	Placebo	1.22 ± 0.28	1.28 ± 0.30	0.374
	LC-Plasma	1.30 ± 0.35	1.36 ± 0.34	0.410
Other genes				
*OVOL1*	Placebo	1.07 ± 0.75	0.81 ± 0.40	0.03 *
	LC-Plasma	1.03 ± 0.64	0.94 ± 0.78	0.543

Mean ± S.D., ^†^: *p* < 0.10, *: *p* < 0.05, **: *p* < 0.01.

**Table 6:** Score changes of skin diagnosis.

Indices	Group	0W	8W	*p* Value
Dryness	Placebo	0.41 ± 0.78	0.88 ± 0.94	0.003 **
	LC-Plasma	1.18 ± 1.27	1.32 ± 1.04	0.503
Erythema	Placebo	1.71 ± 0.87	1.44 ± 1.05	0.107
	LC-Plasma	2.03 ± 0.90	1.71 ± 1.12	0.041 *
Scale	Placebo	0.56 ± 0.79	0.85 ± 0.93	0.110
	LC-Plasma	1.21 ± 1.20	1.21 ± 1.07	0.908
Irritaion	Placebo	0.00 ± 0.00	0.03 ± 0.17	1.000
	LC-Plasma	0.06 ± 0.34	0.00 ± 0.00	1.000
Itching	Placebo	0.00 ± 0.00	0.03 ± 0.17	1.000
	LC-Plasma	0.03 ± 0.17	0.03 ± 0.17	1.000

Mean ± S.D., *: *p* < 0.05, **: *p* < 0.01.

**Table 7:** Changes of the skin indices.

Indices	Group	0W	8W	*p* Value
TEWL (gm^−2^h^−1^)	Placebo	16.85 ± 6.26	21.34 ± 6.80	0.002 **
	LC-Plasma	16.44 ± 5.07	22.09 ± 8.86	0.000 **
Skin Moisture (A.U.)	Placebo	58.32 ± 10.43	55.16 ± 11.79	0.140
	LC-Plasma	55.38 ± 12.13	52.93 ± 15.61	0.200
L*	Placebo	64.51 ± 2.36	65.29 ± 2.11	0.002 **
	LC-Plasma	64.36 ± 2.94	65.33 ± 2.64	0.001 **
a*	Placebo	7.90 ± 1.76	7.82 ± 1.62	0.526
	LC-Plasma	7.72 ± 1.74	7.58 ± 1.42	0.700
b*	Placebo	16.55 ± 1.98	15.97 ± 2.16	0.004 **
	LC-Plasma	16.45 ± 2.02	15.75 ± 2.21	0.000 **
Melanine	Placebo	0.92 ± 0.16	0.87 ± 0.14	0.000 **
	LC-Plasma	0.91 ± 0.20	0.85 ± 0.18	0.000 **
Hb index	Placebo	1.18 ± 0.28	1.08 ± 0.25	0.735
	LC-Plasma	1.16 ± 0.24	1.14 ± 0.21	0.637
HbSO_2_	Placebo	52.58 ± 4.27	56.06 ± 5.02	0.000 **
	LC-Plasma	53.66 ± 5.05	57.95 ± 4.59	0.000 **

Mean ± S.D., **: *p* < 0.01.

## Data Availability

Data is contained within the article.
